# Telephone Fracture Clinic Consultations: A Satisfactory Solution in Lockdown

**DOI:** 10.7759/cureus.14371

**Published:** 2021-04-08

**Authors:** Selina Graham, Emilie Lostis, Oliver Pearce, Michael Kelly

**Affiliations:** 1 Trauma and Orthopaedics, Southmead Hospital, Bristol, GBR

**Keywords:** covid 19, coronavirus, patient satisfaction, remote consultation, fracture clinic, united kingdom

## Abstract

Introduction

The COVID-19 pandemic was a catalyst to many learning opportunities within clinical practice in the UK. Attempts were made to reduce footfall within all institutions and within the study unit; this led to alterations in fracture clinic provision. An alternative method was developed whereby most of the initial contacts were in person and much of the follow-up was done remotely. The aim of this study was to evaluate patient satisfaction and views on this alteration in service.

Methods

The first 299 patients who had fracture clinic appointments delivered by telephone consultation at this institution during the pandemic in early 2020 were retrospectively identified and sent a postal survey. Satisfaction levels were assessed through a degree of agreement with statements (nine items), yes/no answers (four questions), and space for comments.

Results

One hundred and thirty-one survey responses were included (44% response rate). The majority of patients (82%) were satisfied overall with the care they received. Although 78% of patients stated that they preferred a telephone consultation to attend a face-to-face hospital appointment during the pandemic, only 22% stated they would have preferred this in normal (pre-COVID-19) times. Interestingly 62% of patients stated they would be happy for further fracture clinic appointments to be carried out in the same way.

Discussion

Most of the remote consultations were in follow-up rather than new patients. Patients were adaptable to this alternative method of care delivery. There could be a role for its integration into the options for fracture clinic delivery in the future.

## Introduction

The global COVID-19 pandemic accelerated change in almost all spheres of life and in particular healthcare delivery. NHS England and the British Orthopaedic Association (BOA) published guidance on the management of patients with urgent trauma and orthopaedic conditions [1,2]. The aim was to minimise patients’ risk of COVID-19 through reduced contact with acute hospital services. In accordance with this guidance, the study unit instituted changes to the delivery of fracture clinics.

Virtual fracture clinics (VFC) have been championed by many institutes as a means of dealing with overwhelming demand. Various models of this have been published and although the index units are vociferous about their perceived advantages, the concept has not been found to be universally acceptable. The department of health had considered the VFC model prior to the pandemic but advised against instituting a formal VFC at the beginning of the outbreak.

The fracture clinic service in the study unit was a conventional outpatient-based model that followed National Institute for Health and Care Excellence (NICE) guidelines whereby patients with an acute injury were seen the day following their emergency attendance then followed up as the injury required. The various VFC models that had previously been developed elsewhere had been considered but appeared to confer no advantage to the established service.

It was recognised that with the cessation of elective outpatient services there was an opportunity to again evaluate the fracture clinic service. The primary driver was recognition that there were likely to be conflicting anxieties in the patient population relating to both their injury and the pandemic. Changes were proposed that addressed these anxieties and in addition offered time-critical injury assessment and potentially reduced institutional footfall. The proposal was for telephone screening consultations for both new and follow-up fracture clinic appointments, addressing both the injury and the pandemic-related anxieties.

The primary aim of this study was to evaluate patient satisfaction with the fracture clinic telephone consultation system used in this trust during the COVID-19 pandemic. The secondary aim was to elicit their opinion on this versus the conventional approach.

## Materials and methods

This was a retrospective study of 299 patients who had fracture clinic appointments delivered by telephone consultation between March 18, 2020, and April 25, 2020. Of these, 10% were for new injuries and 90% were follow-up appointments. All patients were aged 16 years or older (range=16-98). These patients were sent a postal satisfaction survey. All non-responders were sent one reminder survey. Anyone referred by the institutional emergency department (or surrounding minor injury units) or who had a follow-up appointment in the study period was included. There were no exclusions. Ethical approval was waived by the local committee. The Standards for Reporting Qualitative Research were applied [3].

Satisfaction is a multifactorial construct and our survey attempted to reflect this. The survey and cover letter were designed by adaptation of the Satisfaction with Outpatient Services (SWOPS) questionnaire and the Outpatient Experiences Questionnaire (OPEQ) [4,5]. To the best of our knowledge, no validated tool exists in the literature to facilitate the assessment of patient satisfaction following telephone consultations. The adapted survey was reviewed by two orthopaedic surgeons and six lay people and approved within this study institution for dissemination. The survey consists of nine statements for the patient to rank on a five-point Likert scale, followed by four further questions with yes/no answers and finally a space for further comments [6].

Responses were manually transcribed into a secure electronic database. Data were analysed using Excel (Microsoft Corporation, version 16) pivot tables. Chi-squared statistical analysis by age group (categorical) was performed using the SciPy [7]. A significance level was set at a P-value <0.05 using confidence intervals (CI) of 95%.

The service

All patients who were offered an appointment were contacted. Their injury and the need/desire to attend a fracture clinic appointment were discussed. Completion of the episode as a remote consultation or as a further attendance was then agreed as well as the further management parameters required by the injuries and the anxieties around this. All patients, on completion of any consultation, were offered an open appointment that could either be remote or an in-person event.

## Results

A total of 137 survey responses were collected. After exclusions for incomplete data, 131 surveys were available for analysis, corresponding to a 44% response rate. Almost half (49%) of respondents had lower limb injuries and 51% had upper limb injuries. The majority (85%) of the telephone consultations were for follow-up appointments, the other 15% (n=19) were for new patients.

**Figure 1:**
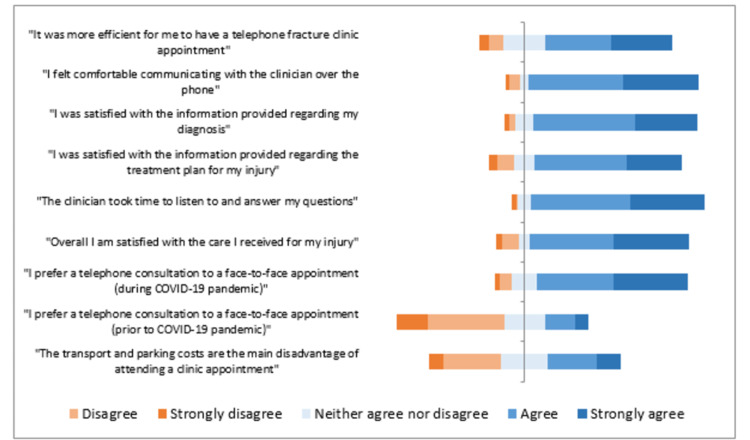
Part 1 survey responses (graphical representation)

Eighty-two percent (n=108) of patients were satisfied overall with the care they received (Figure 1). Telephone consultations were felt to be more efficient by 66% (n=86) of patients (Table 1). The majority of patients (89%) felt comfortable communicating with the clinician over the telephone and felt that the clinician took time to listen to and answer their questions. Most patients would not prefer video calling (73%, n=96; Table 2).

**Table 1:** Part 1 survey responses (percentages)

	Strongly disagree	Disagree	Neither agree nor disagree	Agree	Strongly agree
"It was more efficient for me to have a telephone fracture clinic appointment"	5%	8%	22%	34%	31%
"I felt comfortable communicating with the clinician over the phone"	2%	6%	4%	50%	39%
"I was satisfied with the information provided regarding my diagnosis"	2%	3%	9%	53%	32%
"I was satisfied with the information provided regarding the treatment plan for my injury"	5%	8%	11%	48%	28%
"The clinician took time to listen to and answer my questions"	2%	1%	7%	52%	38%
"Overall I am satisfied with the care I received for my injury"	3%	8%	6%	43%	39%
"I prefer a telephone consultation to a face-to-face appointment (during COVID-19 pandemic)"	2%	6%	14%	40%	38%
"I prefer a telephone consultation to a face-to-face appointment (prior to COVID-19 pandemic)"	17%	39%	21%	16%	7%
"The transport and parking costs are the main disadvantage of attending a clinic appointment"	8%	30%	24%	25%	13%

**Table 2:** Part 2 survey responses (percentages)

	Yes	No
"Would you be happy for further fracture clinic appointments to be carried out in the same way?"	62%	38%
"Would you prefer video calling instead of voice calling?"	29%	71%
"Have you needed to contact us again for further advice?"	17%	83%
"Have you sought further advice from any other sources?"	27%	73%

**Figure 2:**
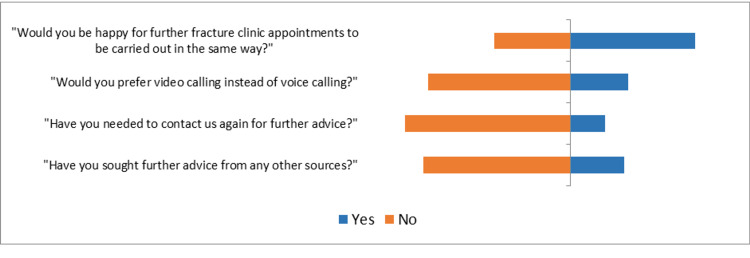
Part 2 survey responses (graphical representation)

The satisfaction rate with regards to the information provided about the diagnosis was 85% (n=111) and with regards to the information provided about the treatment plan was 76% (n=100). Only 17% (n=22) have needed to contact us again for further advice, while 27% (n=35) sought advice from other sources (Figure 2). Of the total 48 patients who sought further advice, 12 required further face-to-face appointments (some of these after a further remote consultation), and 13 used their ‘open appointment’ to arrange attendance at a further consultation in the hospital.

The majority (78%, n=102) preferred a telephone consultation to a face-to-face appointment during the COVID-19 pandemic. Although, only 22% (n=29) stated this preference would have been held in ‘normal’ (pre-COVID-19) times, 62% (n=80) of patients would be happy for telephone fracture clinic appointments in the future, having experienced them. When attending face-to-face appointments, transport and parking costs were not a significant factor for most patients (60%, n=79).

The age groups for analysis were selected as the inter-quartile range, with 25 patients in the <45 age group, 79 patients in the 46-75 age group, and 27 in the >75 group. There was no statistically significant difference in response nature when stratifying against categorical age and appointment type.

## Discussion

This study demonstrated an alternative and effective use of remote consultation technologies. This was used to manage 15% of new referrals and 85% of follow-ups during the pandemic. Only 22% of patients stated they would have preferred a telephone consultation prior to the COVID-19 pandemic, but having experienced this, 62% said then said that they would be happy to receive care in this manner in the future. These data suggest that there is learning from the COVID-19 pandemic that suggests an alternative model to VFCs that has high levels of satisfaction and resilience. The open appointment system remains important because 37% then sought further advice after the telephone consultation and of these, 27% had a further face-to-face consultation.

Prior to the pandemic, an average of 250-300 patients were reviewed in the study unit fracture clinic each week. All reviews were carried out in person and patients were routinely reviewed within 72-hours of referral, in line with previous BOAST guidance [8]. The performance against this target suggests that the local guidance to the feeding emergency departments and minor injury units was adequate and the system was not overwhelmed with patients. VFCs have been championed in many centres quoting unmanageable patient numbers. Models of VFC delivery vary across the UK. The concept most quoted was that first proposed by the Glasgow Royal Infirmary Group following extensive preparatory work and the introduction of a comprehensive selection of injury-specific guidelines [9]. In contrast, other hospitals proposed VFCs predominantly as a triaging service. Studies of VFCs and other forms of teleconsultation in orthopaedics have shown high levels of patient satisfaction [10-15]. There is, however, some evidence for a preference for face-to-face consultations over telephone consultation [16].

When developing the study unit pandemic service, consideration was given to a number of models through which some or all care could be delivered remotely. It was considered important to maintain a service in which decision-making and delivery of care were performed by senior orthopaedic clinicians. Telephone consultations coincided with a number of other changes in practice, including a more consistent application of evidence-based non-operative treatment strategies, senior decision-making at the first point of contact, using absorbable sutures, and increasing the use of removable casts/splints/braces, in accordance with BOA and NHS England guidance [1,2]. The new referrals were predominantly seen face-to-face with only 15% electing to have remote only consultations. The patient satisfaction surveys were undertaken to assess whether the changes met the needs and expectations of patients. Satisfaction outcomes were distributed evenly across age groups and appointment types, suggesting a balanced approach. Despite this, it was interesting to note that although only 22% of patients stated they would have preferred a telephone consultation prior to the COVID-19 pandemic, having then had the experience of this, 62% said they would be happy to receive care in this manner in future. This suggests that remote clinic appointments probably have a role in fracture clinics albeit that this can be quite different from the previously published VFC triaging type models. 

The results, though largely positive, do demonstrate the ongoing need for face-to-face appointments with 37% seeking further advice after the telephone consultation and 27% having had a further in-hospital consultation. Any future re-configuration would also need to take into consideration staff satisfaction. This was not undertaken as part of this study.

There were some notable limitations to this study. First, the study design is susceptible to recall bias as the questionnaires were sent out after the appointments. Second, the time interval between the telephone consultation and receipt of the survey was variable (range 5-39 days, mean 21 days). Third, over half of the questionnaires were either not returned or were incomplete and therefore not available for inclusion in the analysis, leading to the possibility of a representation bias. However, our response rate is as expected for a postal survey [17]. The outcome following the injury may also have impacted satisfaction rates and was not measured. In further work, it would be of interest to consider whether injury severity affected patient satisfaction and to investigate the factors associated with patients requiring face-to-face appointments after a telephone consultation.

## Conclusions

Many aspects of trauma care still require face-to-face attendance, but the data presented here suggest that there is likely to be a role for remote consultations particularly amongst the follow-up population. This would reduce the physical footprint in the clinics and may also reduce the use of hospital resources such as radiology and plaster technician time. The data suggest that when offered alongside a conventional in-hospital clinic service, satisfaction rates are high amongst patients.
